# Fine Mapping of a Novel Major Quantitative Trait Locus, *qPAA7*, That Controls Panicle Apical Abortion in Rice

**DOI:** 10.3389/fpls.2021.683329

**Published:** 2021-07-07

**Authors:** Xiaolei Wang, Lingfeng Li, Xiaotang Sun, Jie Xu, Linjuan Ouyang, Jianmin Bian, Xiaorong Chen, Weixing Li, Xiaosong Peng, Lifang Hu, Yicong Cai, Dahu Zhou, Xiaopeng He, Junru Fu, Haihui Fu, Haohua He, Changlan Zhu

**Affiliations:** Key Laboratory of Crop Physiology, Ecology and Genetic Breeding, Ministry of Education, College of Agronomy, Jiangxi Agricultural University, Nanchang, China

**Keywords:** rice, panicle apical abortion, fine mapping, quantitative trait locus, CSSL

## Abstract

The panicle apical abortion (PAA) causes severe yield losses in rice production, but details about its development and molecular basis remain elusive. Here, we detected PAA quantitative trait loci (QTLs) in three environments using a set of chromosome segment substitution lines (CSSLs) that was constructed with *indica* Changhui121 as the recurrent parent and *japonica* Koshihikari as the donor parent. First, we identified a novel major effector quantitative trait locus, *qPAA7*, and selected a severe PAA line, CSSL176, which had the highest PAA rate among CSSLs having Koshihikari segments at this locus. Next, an F_2_ population was constructed from a cross between CSS176 and CH121. Using F_2_ to make recombinantion analysis, *qPAA7* was mapped to an 73.8-kb interval in chromosome 7. Among nine candidate genes within this interval, there isn’t any known genes affecting PAA. According to the gene annotation, gene expression profile and alignment of genomic DNA, *LOC_Os07g41220* and *LOC_Os07g41280* were predicted as putative candidate genes of *qPAA7*. Our study provides a foundation for cloning and functional characterization of the target gene from this locus.

## Introduction

Rice is the staple food of half of the world’s population. A high, stable yield of rice has always been one of the most important goals pursued by breeders. The rice yield is mainly determined by the number of panicles per unit area, grain number per panicle and 1,000-grain weight (Xing and Zhang, 2010). Increasing the grain number per panicle is a prior goal of many rice breeders in high-yield breeding. The panicle architecture, which is characterized by its size and branching pattern, determines the number of spikelets, and then number of grains per panicle (Sakamoto and Matsuoka, 2004). Large panicle with more branches and spikelets have been preferred in breeding programs for new rice types with higher yield. Thus, understanding the molecular genetic mechanisms of panicle development and identification of superior alleles for large panicles are of great interest to both plant biologists and plant breeders.

Rice panicles are composed of main axis, primary branches, secondary branches and spikelets (Itoh et al., 2005). In rice, panicle development can be roughly divided into two main stages. First, when vegetative meristems changed to the reproductive meristems, the shoot apical meristem (SAM) transformed into an inflorescence meristem (IM), which further initiates the primary branch meristems (BMs) and forms the main axis of the inflorescence. Subsequently, the primary BMs produce secondary BMs and spikelet meristems (SMs). SMs are also initiated from the secondary BMs and finally form spikelets (Komatsu K. et al., 2003; Ikeda et al., 2004). In most of the studies, spikelet degeneration has been reported during the panicle elongation stage (Ali et al., 2019). For example, invasion of any stress during meiosis, could block the development of floral organs that results in spikelet degeneration. In a practical sense, early stage degeneration of spikelets is a detrimental factor for the final yield, which could result in 3–50% yield loss in a single panicle (Yamagishi et al., 2004; Cheng et al., 2011; Li et al., 2018).

Over the past two decades, a number of genes regulating panicle development have been identified and functionally characterized (Vollbrecht et al., 2005; Teo et al., 2014; Zuo and Li, 2014). For example, *LAX PANICLE1* (*Lax1*) (Komatsu K. et al., 2003; Tetsuo and Junko, 2009); *Lax2* (Tabuchi et al., 2011) and *MONOCULM1* (*MOC1*) (Li et al., 2003) are important regulatory factors in axillary meristem formation in rice. *LAX PANICLE (LAX)* gene controls early developmental switches involved in the initiation of axillary meristems and branch formation in rice (Komatsu K. et al., 2003). *ABERRANT PANICLE ORGANIZATION 1* (*APO1*) (Ikeda et al., 2005, 2007), *LEAFY* (*RFL*)/*APO2* (Rao et al., 2008) and *FRIZZY PANICLE* (*FZP*) (Komatsu M. et al., 2003; Zhu et al., 2003) are involved in maintaining the identity of primary branch meristems by preventing precocious conversion of primary branch meristems to spikelet meristems. In addition, *GRAIN-NUMBER1* (*Gn1a*) is a gene for cytokinin oxidase/dehydrogenase (*OsCKX2*), an enzyme that degrades the phytohormone cytokinin. Reduced expression of *OsCKX2* causes cytokinin accumulation in inflorescence meristems and increases the number of reproductive organs, resulting in enhanced grain yield (Ashikari et al., 2005). These genes mainly play a role in the initiation and transformation stages of panicle development. Elucidating the functions of these genes and their regulatory relationships will contribute to breeding new elite rice cultivars with “ideal” plant architecture and higher grain yield.

During the growth and development of rice, panicle abortion frequently occurs at either the top or basal parts of the panicle, especially under adverse climatic conditions (Yao et al., 2000; Tan et al., 2011). Panicle apical abortion (PAA) is very unfavorable for the formation of large panicles. PAA quantitative trait loci (QTLs) have been detected on chromosomes 1–11 (Xu et al., 2007; Cheng et al., 2011; Tan et al., 2011; Wang et al., 2011; Zhang et al., 2017). However, only a few of QTLs have been fine mapped. For example, *qPAA8* was fine mapped between RM22476-8-IN112 markers on chromosome 8 in an interval of approximately 37-kb (Cheng et al., 2011), and *qPAA3* was fine mapped between M6929-RM1319 markers on chromosome 3 in an interval of approximately 97.3-kb (Zhang X.Y. et al., 2015). So far, QTL of PAA has not been successfully cloned. In recent years, several PAA genes have been cloned using the PAA mutants of rice (Bai et al., 2015; Heng et al., 2018; Peng et al., 2018; Wang et al., 2018; Zafar et al., 2020). For example, Mutation in *TUTOU1*, which encodes a SCAR-like protein modulating actin organization, causes a pleiotropic phenotype including panicle apical abortion (Bai et al., 2015). *OsALMT7* is reported as a malate transporter that plays its role in the development of panicle apical portions (Heng et al., 2018). The function of *OsCIPK31* is disrupted due to excessive accumulation of reactive oxygen species (ROS), leading to cell death in rice panicle (Peng et al., 2018). *SQUAMOSA PROMOTERBINDING PROTEIN-LIKE 6* (*SPL6*) represses signaling outputs of endoplasmic reticulum (ER) stress in control of panicle cell death in rice (Wang et al., 2018). *DEGENERATED PANICLE AND PARTIAL STERILITY 1* (*DPS1*) encodes a cystathionine β-synthase domain containing protein that plays a vital role in regulating ROS homeostasis, anther cuticle formation, and panicle development in rice (Zafar et al., 2020). Despite this progress, the molecular and genetic mechanisms underlying PAA are still poorly understood.

In this study, a set of 132 CSSLs was used to detect the QTLs of PAA under three environments, aiming to identify a novel major QTLs/genes of PAA and clarify the mechanism of PAA genetic regulation, which provides a theoretical basis for breeding high-yielding rice varieties.

## Materials and Methods

### Plant Materials and Planting Conditions

A set of CSSLs was constructed by using Changhui 121 (CH121) and Koshihikari as parental lines that included 132 lines at BC_3_F_8_, BC_4_F_7_ or BC_5_F_6_. The recurrent parent CH121 is an elite rice restorer line breeding developed by our laboratory (He et al., 2008). The donor parent Koshihikari is a Japanese *japonica* variety. To fine map the major locus *qPAA7*, a severe PAA line, CSSL176, which harbors the *qPAA7* allele, was selected for backcrossing with the recurrent CH121 parent. Derived F_2_ CSSL176/CH121 populations were subsequently constructed by self-pollination.

The test environments for the two parents and 132 CSSLs are shown in Table 1. Each family was represented by three rows of ten plants that were planted in a randomized block design. CH121, CSSL176 and F_2_ were planted as middle-season rice and late-season rice in Nanchang, Jiangxi (N 28.45°, E 115.50°) in 2017 and 2018, respectively, and CH121, CSSL176 and F_2_ were planted in Sanya, Hainan (N 18.14°, E 109.31°) in 2019, with a spacing of 17 × 22 cm. Crop management and the control of diseases and insect pests were performed as locally recommended.

**TABLE 1:** Test environments of the 132 CSSLs, CH121 and Koshihikari.

Environment	Replication	Crop location	Crop season
E1	2	Nanchang, Jiangxi N 28.45°, E115.50°	May-Oct 2016
E2	2	Nanchang, Jiangxi N 28.45°, E115.50°	Jun-Nov 2016
E3	2	Sanya, Hainan N18.14°, E109.31°	Dec 2016-May 2017

### Phenotypic Evaluation

At the rice heading stage, the aborted spikelets rate was investigated to CSSL176/CH121 F_2_ individuals, and five plants that were randomly selected from the parents and 132 CSSLs, respectively. The aborted spikelets rate was calculated according to the following equation: aborted spikelets rate (%) = (PAA spikelet number/total spikelet number)^∗^100.

To investigate the occurring time of PAA during panicle development, we divided the developmental course of panicles into eight stages according to the panicle length (∼1, 4, 7, 10, 13, 16, 20, and 24 cm).

### Marker Analysis

Genomic DNA was extracted from fresh leaves by using CTAB method (Rogers and Bendich, 1985). The PCR reaction mixture (total volume of 10 μL) (Panaud et al., 1996), contained 1 μL 10 × buffer (Mg^2+^ Plus), 0.2 μL dNTPs, 0.5 μL forward primer (10 μmol/L), 0.5 μL reverse primer (10 μmol/L), 2 μL DNA, 5.7 μL ddH_2_O_2_ and 0.1 μL Taq polymerase (5 U/μL). The reaction cycles were as follows: One initial step (94°C, 5 min), 34 cycles (94°C, 30 s; 55–58°C, 30 s; 72°C, 30 s), a final extension step (72°C, 10 min) and followed by storage at 4°C. The PCR products were subjected to electrophoresis on 8% polyacrylamide gel and silver staining for visualization.

The simple sequence repeat (SSR) primers and sequence-tagged site (STS) were designed according to sequences from the Gramene database^1^. The insertion/deletion (InDel) markers were designed using Primer 5.0 according to the CH121 and CSSL176 resequencing data.

### Construction of Genetic Linkage Map

According to the genotype of 132 CSSLs using 125 SSR and 17 STS markers, the original data set was constructed. “1” indicates Koshihikari genotype; “2” represents CH121 genotype; “H” indicates heterozygote; “0” denotes an unknown genotype (Supplementary Figure 1). The linkage map was constructed by MapMaker/EXP 3.0 (Lander et al., 1987) software with the default parameter. The main procedures were as follows: First, the linkage groups were calculated through two-point method, and “GROUP” was used to infer the optimal linkage groups. Second, the framework structure of genetic linkage map was constructed using multi-point analysis, and “COMPARE” was used to sorted SSR/STS markers. The recombination frequencies were converted into map distances (cM) using the function of Kosambi (1944). Finally, according to the map distances among markers, Mapchart 2.1 (Voorrips, 2002) was used to draw the genetic linkage map.

### qRT-PCR Analysis

Total RNA was extracted using the TaKaRa MiniBEST Universal RNA Extraction Kit (TaKaRa, China). qRT-PCR was carried out using an ABI7500 fast real-time PCR system with the SYBR Premix Ex Taq (TaKaRa; RR041A), following the manufacturer’s instructions. The *OsActin* gene was used as an internal control. The gene expression differences were estimated using the 2^–Δ^ ^Δ^ ^Ct^ method (Livak and Schmittgen, 2001). Three biological and three technical repeats were performed in the experiments. The information of primers used in the qRT-PCR analysis is listed in Supplementary Table 1.

### Data Analysis and QTL Mapping

QTL analysis was performed using the IciMapping 4.1 software (Wang et al., 2006; Meng et al., 2015), and a logarithm-of-odds (LOD) score of 2.5 was chosen as a threshold for determining QTLs of the traits. Data analysis was performed using the one-way analysis of variance (ANOVA) module within Statistical Package for Social Sciences 17.0 (SPSS 17.0). Statistical significance was set at an alpha level of *P* < 0.05.

## Results

### Aborted Spikelets Rate of Parents and CSSL

**FIGURE 1:**
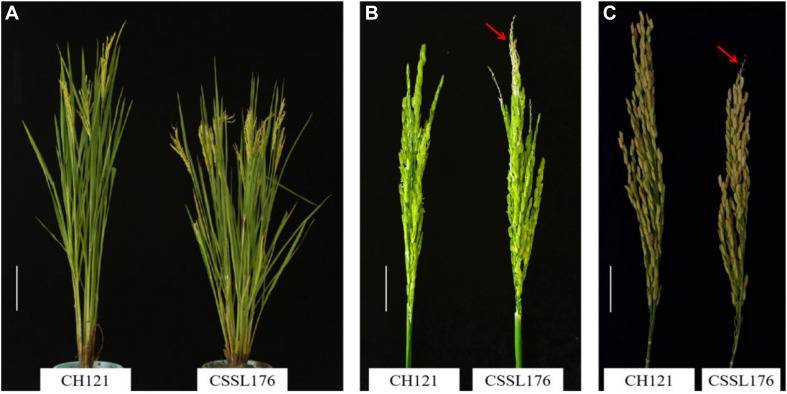
Phenotypes of the CSSL176 and CH121. **(A)** Plants at the heading stage, Bar = 16 cm. **(B)** Panicles at the heading stage. Bar = 4 cm. **(C)** Panicles at maturity period, Bar = 3 cm. The arrows show the PAA in CSSL176.

The aborted spikelets rate of 132 CSSLs were investigated in three environments. Relative to the recurrent parent CH121, 8 CSSLs show the aborted spikelets at the apical portion of panicle under three environments, but the other 124 CSSLs did not show aborted spikelets. The aborted spikelets rates of 8 CSSLs were significantly higher than CH121 (Table 2). Comparing with the others lines, CSSL176 had the highest aborted spikelets rates, i.e., 9.05, 14.65 and 18.85% in three environments, with a mean aborted spikelets rate of 14.18% (Figure 1).

**TABLE 2:** Aborted spikelets rate of CH121, Koshihikari and 8 CSSLs.

Trait	Aborted spikelets rate (%)									
	
Lines	CH121	Koshihikari	CSSL15	CSSL143	CSSL145	CSSL176	CSSL200	CSSL205	CSSL207	CSSL208
E1	0.00 ± 0.00	0.00 ± 0.00	5.40 ± 0.10**	3.45 ± 0.25**	3.95 ± 0.15**	9.05 ± 0.15**	3.40 ± 0.05**	4.85 ± 0.10**	9.70 ± 0.30**	6.60 ± 0.30**
E2	0.00 ± 0.00	0.00 ± 0.00	6.25 ± 0.15**	4.40 ± 0.10**	4.00 ± 0.30**	14.65 ± 0.25**	2.80 ± 0.10**	4.50 ± 0.20**	9.80 ± 0.10**	7.30 ± 0.20**
E3	0.00 ± 0.00	0.00 ± 0.00	7.20 ± 0.10**	5.30 ± 0.10**	4.50 ± 0.10**	18.85 ± 0.25**	3.50 ± 0.30**	5.40 ± 0.25**	10.60 ± 0.30**	7.70 ± 0.15**

**“^∗∗^” represent P < 0.01.**

### PAA QTL Detection

**FIGURE 2:**
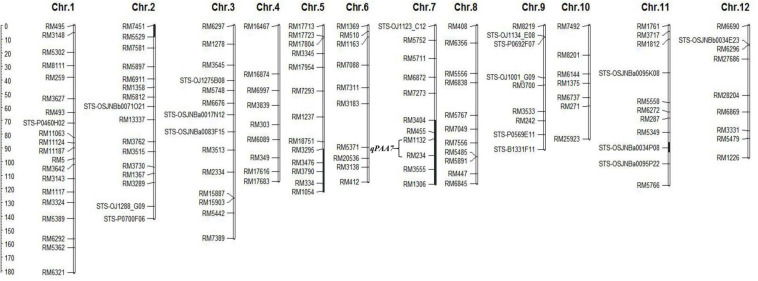
Linkage map of CSSL176. The black bars represent the Koshihikari chromosomal segments in CSSL176. The scale of the ruler indicates the genetic distance, the unit “cM”.

In previously study, a set of CSSLs was constructed in our laboratory (Wang et al., 2020b). To identify genes controlling PAA, we conducted a QTL analysis for PAA using 132 CSSLs population. Genotyping was performed using 142 molecular markers, including 125 SSR markers and 17 STS markers. The linkage map of SSR/STS markers was shown in Figure 2.

In total, ten QTLs of PAA were identified using 132 CSSLs under three environments, and mapped to seven chromosomes (Table 3). Among them, *qPAA3*, *qPAA4.1* and *qPAA7* were detected in all three environments, with an average phenotypic variation explained (PVE) of 4.26, 6.85 and 26.96%, respectively. The others QTL, *qPAA1.1*, *qPAA1.2*, *qPAA4.2*, *qPAA5.1*, *qPAA5.2*, *qPAA9*, and *qPAA11* were detected in one or two environments, with PVE of 1.38–12.52%. PVE of *qPAA7* is relatively large, with the positive allele from Koshihikari.

**TABLE 3:** Detected QTLs affecting PAA traits using CSSL population across three environments.

QTL^a^	Chr.	Marker^b^	Environment	LOD^c^	PVE(%)^d^	Add^e^
*qPAA1.1*	1	RM8111-RM259	E1	4.36	1.88	0.61
			E3	6.58	1.38	0.48
*qPAA1.2*	1	RM3143-RM5302	E3	15.62	3.89	–2.08
*qPAA3*	3	RM3513-RM2334	E1	12.02	5.97	–1.17
			E2	3.84	2.25	–0.69
			E3	17.67	4.57	–0.94
*qPAA4.1*	4	RM17616-RM17683	E1	11.23	5.61	–5.48
			E2	15.92	8.07	–5.29
			E3	19.14	6.86	–6.4
*qPAA4.2*	4	RM16467-RM16874	E3	9.81	2.19	0.79
*qPAA5.1*	5	RM3295-RM3476	E1	13.46	6.87	1.76
			E2	16.82	12.52	2.27
*qPAA5.2*	5	RM334-RM1054	E3	12.79	8.50	1.89
*qPAA7*	7	RM1132-RM234	E1	41.28	36.89	2.55
			E2	27.98	25.95	2.04
			E3	42.23	18.04	1.63
*qPAA9*	9	STS-OJ1001_G09-RM3700	E3	25.93	7.89	–1.23
*qPAA11*	11	RM5349-STS-OSJNBa0034P08	E3	25.78	7.82	1.34

*^*a*^The QTLs for PAA are temporarily named “qPAA + number of chromosome + number of QTL”.*

*^*b*^Flanking markers of PAA-related QTLs.*

*^*c*^Peak LOD value of the QTL.*

*^*d*^The phenotypic variation explained by the putative QTL.*

*^*e*^Additive effect. Positive, negative additive effects: increased, reduced PAA from Koshihikari allele, respectively.*

### Further Localization of *qPAA7*

As mentioned above, *qPAA7* was detected between markers RM1132-RM234 in three environments. Among 132 CSSLs, CSSL15, CSSL176, CSSL205, CSSL207 and CSSL208 lines harbors *qPAA7* and showed aborted spikelets at the apical portion of panicle at the heading stage (Table 4). CSSL176 was selected according to its graphical genotype and phenotypic performance, then backcrossed with CH121. A segregating F_2_ population was constructed to identify the existence of *qPAA7*. Four substituted fragments from the donor parent Koshihikari, distributed on chromosomes 2, 5, 7, and 11, covered by 16 SSR markers (Figure 2).

**TABLE 4:** Graphical genotypes and aborted spikelets rate of 8 CSSLs.

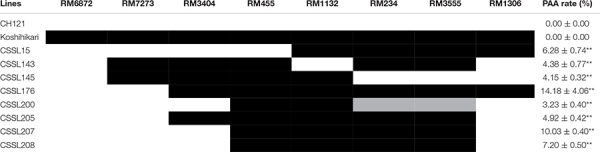

**“White grid” represents the genotype of CH121. “Black grid” represents the genotype of Koshihikari. “Gray grid” represents the genotype of heterozygous. “^∗∗^” represent P < 0.01.**

**FIGURE 3:**
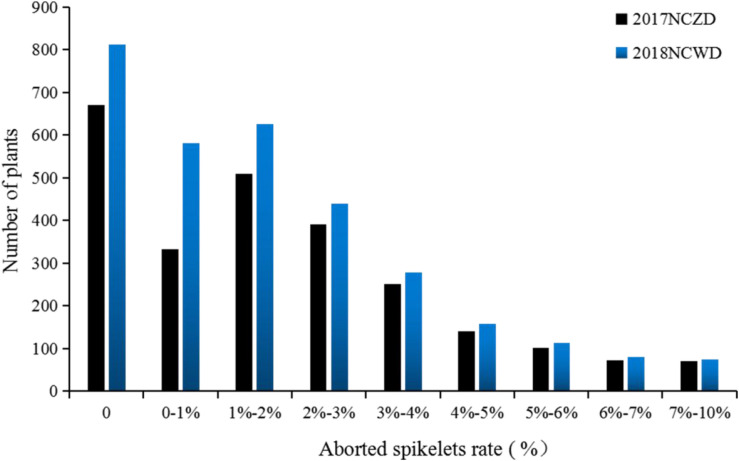
The severity of panicle apical abortion (PAA) distribution of F_2_ plants from the cross of CSSL176 × CH121. 2017NCZD and 2018 NCWD represent the middle-season rice in Nanchang, Jiangxi in 2017 and the late-season rice in Nanchang, Jiangxi in 2018, respectively.

We surveyed the aborted spikelets rates of different F_1_ and F_2_ populations as middle-season rice in Nanchang, Jiangxi, in 2017 and as late-season rice in Nanchang, Jiangxi, in 2018. The results indicated that F_1_ showed the phenotype of PAA, and F_2_ populations presented a skewed distribution (Figure 3). However, the individual-plant aborted spikelets rate of F_2_ was between those of CSSL176 and CH121. We speculated that the trait of PAA was controlled by a semidominant gene. Next, thirty plants were selected from F_2_ in 2017 middle-season rice and 2018 late-season rice, respectively, to generate high-PAA and non-PAA bulks, each consisting of fifteen plants. The high-PAA and non-PAA bulks were amplificated with the 16 SSR molecular markers that came from the donor parent Koshihikari and distributed on chromosomes 2, 5, 7 and 11. It was found that 6 SSR molecular markers were polymorphic at the end of chromosome 7 (RM3404, RM455, RM1132, RM234, RM3555, and RM1306).

### Fine Mapping of *qPAA7*

**FIGURE 4:**
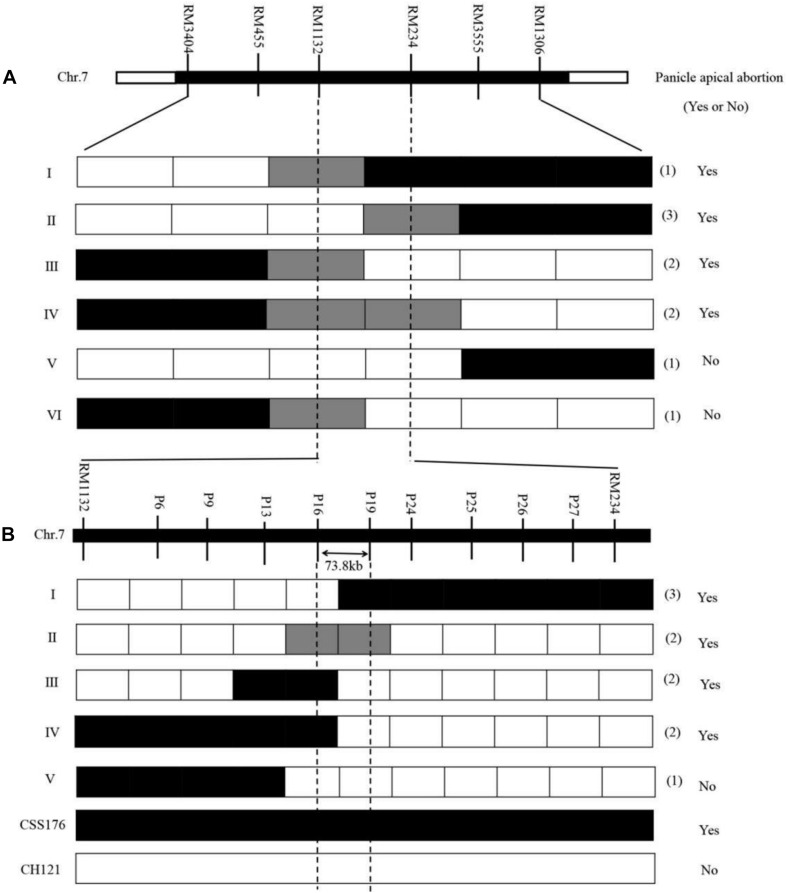
Fine mapping of *qPAA7*. **(A)** Linkage analysis of phenotypes and marker genotypes between RM1132 and RM234. **(B)** Linkage analysis of phenotypes and marker genotypes between P16 and P19. Panicle apical abortion (Yes or No), represents the panicle apical abortion, and no panicle apical abortion, respectively.

The CSSL176/CH121 backcrossed generations were applied to finely map *qPAA7*. For the first round of fine mapping. Nine recombinants were identified from 300 F_2_ individuals using six markers (RM3404, RM455, RM1132, RM234, RM3555 and RM1306), which were located in the target region and were polymorphic between “CH121” and “CSSL176.” Nine recombinants were divided into six genotypes (I–VI), and among them, recombinants I, II, and V confirmed the right border of the region of interest, whereas recombinants III, IV, and VI confirmed the left border (Figure 4A). The genotypic and phenotypic analysis of the recombinants narrowed the *qPAA7* region to the segment between markers RM1132 and RM234, which corresponded to a physical distance of 1.48 Mb.

For the second round of fine mapping. The genotypes of the 3152 F_2_ individuals were analyzed by using the polymorphism markers RM1132 and RM234, and 24 residual heterozygous lines (RHLs) were found. The selfing of these RHLs generated a new segregating F_2_ population (from the heterozygote F_1_ for the target chromosomal segment) that contained 2100 individuals, which were evaluated for *qPAA7* and rescreened using nine polymorphisms InDel markers. The InDel markers were located within the target region (Supplementary Table 2). Based on the genotypic and phenotypic analysis, the *qPAA7* region was narrowed to the segment flanked by markers P11 and P19 (Figure 4B). This new region corresponded to a 73.8-kb segment in the “Nipponbare” reference genome.

### Candidate Gene Analysis

According to the Nipponbare genome annotation^2^. Nine genes were annotated within the 73.8-kb region of *qPAA7* (Table 5), including five cloned genes (*LOC_Os07g41200*, *LOC_Os07g41210*, *LOC_Os07g41230*, *LOC_Os07g41240* and *LOC_Os07g41250*). Among them, *LOC_Os07g41200* encodes a TONNEAU1-recruiting motif protein, which regulates longitudinal cell elongation (Wang S.K. et al., 2015). *LOC_Os07g41210* is a negative regulator of *GL7* (Wang Y.X. et al., 2015). *LOC_Os07g41230*, which is annotated as methyl esterase-like, affects reactive oxygen species (ROS) accumulation by interacting with thioredoxin *OsTrxm* in rice (Hu et al., 2021). *LOC_Os07g41240* encodes CYP78A13, which regulates cell size in the rice grain embryo (Nagasawa et al., 2013; Yang et al., 2013; Xu et al., 2015). *LOC_Os07g41250* annotated as nitrate and di/tripeptide transporter (NPF) gene family. Studies have shown that the nitrate and di/tripeptide transporter (NPF) have diverse substrates and underlie many biological processes in plants (Ouyang et al., 2010; Léran et al., 2014).

**TABLE 5:** Annotation information of candidate genes identified within *qPAA7*.

Gene ID	Physical location (bp)	Putative function
*LOC_Os07g 41200*	24,669,324–24,664,168	Expressed protein
*LOC_Os07g 41210*	24,682,428–24,682,874	Negative regulator of GL7
*LOC_Os07g 41220*	24,685,870–24,687,877	Peptidase aspartic family protein
*LOC_Os07g 41230*	24,699,495–24,703,794	Esterase, putative, expressed
*LOC_Os07g 41240*	24,713,778–24,715,813	Cytochrome P450
*LOC_Os07g 41250*	24,720,067–24,723,873	Peptide transporter PTR2
*LOC_Os07g 41260*	24,724,562–24,728,180	PPR repeat domain containing protein
*LOC_Os07g 41270*	24,734,126–24,734,779	Retrotransposon protein
*LOC_Os07g 41280*	24,736,971–24,742,295	6-phosphogluconolactonase

**FIGURE 5:**
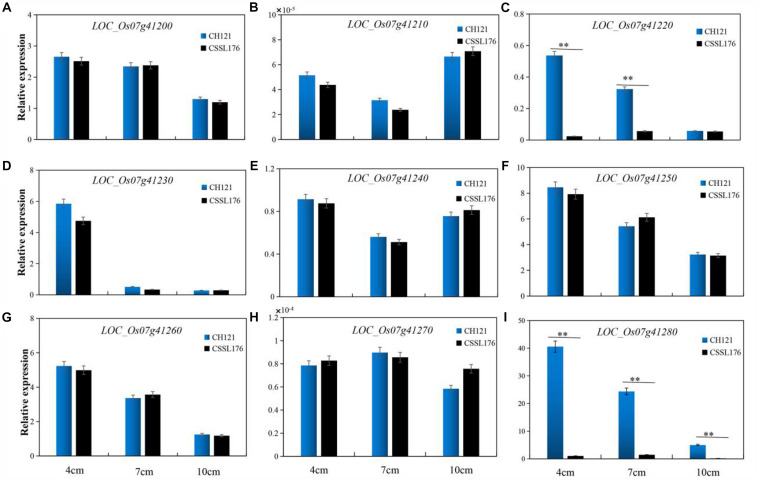
The expression levels of **(A)** *LOC_Os07g41200*, **(B)** *LOC_Os07g41210*, **(C)** *LOC_Os07g41220*, **(D)** *LOC_Os07g41230*, **(E)** *LOC_Os07g41240*, **(F)** *LOC_Os07g41250*, **(G)** *LOC_Os07g41260*, **(H)** *LOC_Os07g41270*, **(I)** *LOC_Os07g41280* between CH121 and CSSL176 in 4 cm, 7 cm, and 10 cm panicles, respectively.

**FIGURE 6:**
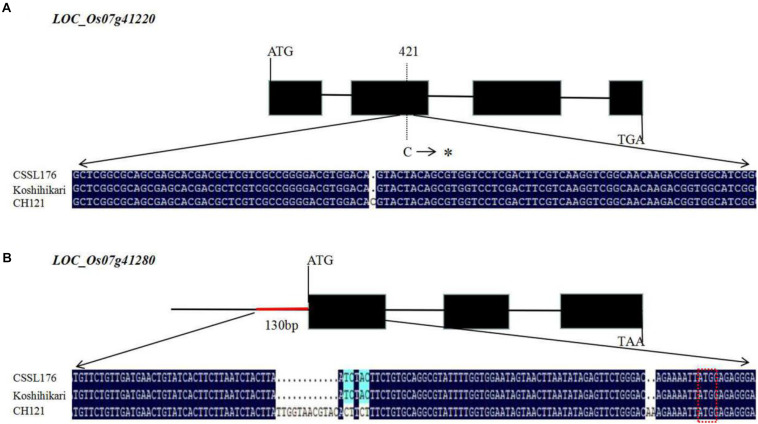
Structure and sequence alignment of candidate genes. **(A)** Gene structure of *LOC_Os07g41220* and sequence differences in *LOC_Os07g41220* between CH121 and CSSL176 **(B)** Gene structure of *LOC_Os07g41280* and sequence differences in *LOC_Os07g41280* between CH121 and CSSL176. The three base sequences in the red box are the start codon. The symbol * means the C deletion.

We observed the spikelets of rice at eight stages according to the panicle length (∼1, 4, 7, 10, 13, 16, 20, and 24 cm) and found that the period of 4–10 cm panicle length is critical for PAA. Therefore, we analyzed the expression levels of the nine candidate genes between CH121 and CSSL176 in samples of 4, 7, and 10 cm panicles. qRT-PCR data analysis indicated that *LOC_Os07g41200*, *LOC_Os07g41210*, *LOC_Os07g41230*, *LOC_Os07g41240*, *LOC_Os07g41250*, *LOC_Os07g41260*, and *LOC_Os07g41270* showed no significant difference between CH121 and CSSL176. *LOC_Os07g41220* showed a significant difference between CH121 and CSSL176 in 4 and 7 cm panicles, but no significant difference between CH121 and CSSL176 in 10 cm panicles. *LOC_Os07g41280* showed significant differences between CH121 and CSSL176 in 4, 7, and 10 cm panicles (Figure 5). To further obtain strong evidence to determine the most promising candidate gene, the genomic DNA sequences of the *LOC_Os07g41220* and *LOC_Os07g41280* were amplified and compared between CH121 and CSSL176. We found that *LOC_Os07g41220* and *LOC_Os07g41280* have different sequences in the CDS region and promoter region, respectively. Among them, *LOC_Os07g41220* had one SNP (C deletion) in the CDS region in CSSL176 compared with CH121 (421 bp downstream from the start codon ATG). *LOC_Os07g41280* had two deletions (9–10 and 70–81 bp upstream from the start codon ATG), together two two-base substitutions at 64–65 and 67–68 bp upstream from the start codon ATG) in the promoter region (Figure 6).

## Discussion

Increasing the number of grains per panicle and cultivating large-panicle-type rice are important ways to further increase rice yield. Normal panicle development is the basis of the large panicle formation. PAA often occurs in agricultural production, reducing the total spikelet number per panicle, and finally causing great yield loss. The aborted spikelets rates of some rice varieties can reach 50–60% under extreme weather conditions (Wang et al., 2017; Ali et al., 2019). Elucidating the genetic mechanism of PAA is helpful for preventing yield loss due to PAA in rice and for cultivating new rice varieties with “ideal” plant architectures and high yields. However, the genetic and molecular mechanisms of PAA are still unclear. *Indica* and *Japonica* are two subspecies of cultivated rice in Asia. Because hybrids between *indica* and *japonica* show strong heterosis, they were widely used in super-high yield rice breeding. *Indica*/*japonica* hybrids have shown prominent heterosis in rice panicle characteristics, such as many rachis branches, relatively stout stems and a large number of grains per panicle. Due to large genetic differentiation between *Indica* and *Japonica* subspecies, PAA often occurs in *indica*/*japonica* hybrid progeny (Yamagishi et al., 2004; Tan et al., 2011). In this study, a set of CSSLs were constructed from Changhui 121 (non-PAA) and Koshihikari (non-PAA), including BC_3_F_8_, BC_4_F_7_, and BC_5_F_6_. Among 132 CSSLs, eight CSSLs showed obvious PAA under three environments. The same phenomenon has been observed in another set of inter-subspecific CSSLs constructed by our laboratory, we speculated that the gene-gene interaction between subspecies lead to the phenomenon of PAA, which has been reported in other traits of rice (Zeng et al., 2009; Sun et al., 2010).

Genetic factors are the direct cause of PAA in rice, while environmental conditions such as temperature, humidity and N nutrition levels in panicle primordium differentiation stage have a great influence on PAA. In this study, we found that the aborted spikelets rate of the 8 CSSLs in winter in Sanya, Hainan, was obviously higher than that in middle-season and late-season in Nanchang, Jiangxi (Table 2), being consistent with Tan et al. (2011). Considering the differences in localities and seasons during panicle primordium differentiation stage, especially the differences in temperature. The results of variance analysis showed that line and experimental environment accounted for 83.86 and 5.76% of the total variation, respectively, and line-environment interaction accounted for 10.61% of the total variation, all of them reached an extremely significant level (Supplementary Table 3). It is shown that the phenotypic differences between parents and CSSLs under the three environments are related to genotype-environment interactions. Low temperature during panicle development stage should be noticed as one of the important unfavorable factors. When the CSSL176 was planted in the winter of Sanya, Hainan, the panicle primordium differentiation stage is from late February to early March. We found that the daily average temperature of February 22nd–23th and March 7th–10th were below 23°C (Supplementary Figure 2), this possibly influences the panicle development of CSSL176 and leads to an increase in the aborted spikelets rate.

PAA have a complex genetic background, and greatly affected by the environment. Therefore, it is difficult to fine map QTLs using the common population types such as F_2_, recombinant inbred lines (RILs) and backcross populations (Qiao et al., 2016). However, CSSL or near-isogenic lines (NILs) from interspecific hybridization are useful for QTL mapping (Wang et al., 2020a) and marker-assisted breeding (Yang et al., 2010). In addition, secondary F_2_ and F_3_ groups can be derived from further backcrossing of selected CSSLs/NILs with the recurrent parent and can be used for the fine mapping and cloning of QTLs (Hu et al., 2018; Zhang et al., 2020). In this study, 132 CSSLs were used to identify the QTL of PAA. Seven QTLs were detected in one or two environments, and the other three QTLs, *qPAA3*, *qPAA4.1* and *qPAA7*, were observed in all three environments. It was found that the QTL for PAA (*qPAA5*) were identified and located in the vicinity of QTL detected in previous reports (Cheng et al., 2011). However, the QTLs/genes of PAA had not been publicly reported in the *qPAA7* interval. Therefore, *qPAA7* is a novel locus that controls PAA in rice.

The higher accumulation of ROS in cells is one of the main reasons for the PAA in rice (Peng et al., 2018; Zafar et al., 2020). Recent studies have shown that ROS is an important signal for gene activation and plays an important role in plant growth and development, biotic and abiotic environmental stimuli responses, and programmed cell death (PCD) (Mittler, 2017). We analyzed the nine gene annotations in the *qPAA7* interval. According to the results of the previous studies, *LOC_Os07g41220*,*LOC_Os07g41230*, and *LOC_Os07g41280* showed the PCD or accumulation of ROS functions, and the other six candidate genes did not show similar functions (Zhang L. et al., 2015; Xiao et al., 2018; Lv et al., 2020). *LOC_Os07g41220* was annotated as a peptidase aspartic family protein, and studies have found that aspartic proteases are related to plant development and cell death (Niu et al., 2013). Therefore, it is speculated that *LOC_Os07g41220* has a similar functions. *LOC_Os07g41230*, which is annotated as methyl esterase-like, affects ROS accumulation by interacting with thioredoxin *OsTrxm* in rice (Hu et al., 2021). *LOC_Os07g41280* is annotated as 6-phosphogluconolactase, and abnormal plant development is observed in Arabidopsis T-DNA insertion mutants (Xiong et al., 2009). In Arabidopsis, 6-phosphogluconolactonase 3 (PGL3) can interact with thioredoxin *Trxm2* in the cytosol, further affecting the redox balance in the cell (Holscher et al., 2014). According to the previous alignment of genomic DNA and qRT-PCR analyze between CH121 and CSSL176, we found that *LOC_Os07g41220* and *LOC_Os07g41280* were most likely the candidate genes of *qPAA7.*

In this study, we identified the QTLs for panicle apical abortion using a total of 132 CSSLs in three environments. A novel major quantitative trait locus, *qPAA7*, was identified, then was fine mapped into an approximate 73.8Kb interval between the P11 and P19 markers on chromosome 7. There are nine candidate genes in *qPAA7* region, among which *LOC_Os07g41220* and *LOC_Os07g41280* are most likely candidate genes of *qPAA7*. Transgenic studies are the gold standard for the validation of candidate gene function. In the future, such studies should be carried out for *LOC_Os07g41220* and *LOC_Os07g41280* to further elucidate their roles.

## Data Availability Statement

The original contributions presented in the study are included in the article/Supplementary Material, further inquiries can be directed to the corresponding author/s.

## Author Contributions

XW and CZ performed the experiments and wrote the manuscript. XS and LO analyzed the phenotypic data. JX, JF, XC, and XP contributed to PCR genotyping. WL, XH, and LL contributed to fled experiments. JB, LH, YC, HF, and DZ performed QTL analysis. HH and CZ designed the experiments. All the authors read and approved the final manuscript and approved the submitted version.

## Conflict of Interest

The authors declare that the research was conducted in the absence of any commercial or financial relationships that could be construed as a potential conflict of interest.

## Footnotes

1 http://www.gramene.org/archive

2 http://rice.plantbiology.msu.edu

## Supplementary Material

Supplementary Figure 1 The genotype of 132 CSSLs.

Supplementary Figure 2 The daily average temperature of the 30-day period from rice booting to heading stage.

Supplementary Table 1 Primers for qRT-PCR in this study.

Supplementary Table 2 Primers for SSR and InDel markers in this study.

Supplementary Table 3 Analysis of variance for aborted spikelets rate of 8 CSSLs in three environments.
