# Supplementary material for: Fine Mapping of a Novel Major Quantitative Trait Locus, qPAA7, That Controls Panicle Apical Abortion in Rice
Source: Front Plant Sci. 2021 Jul 7;12:683329. doi: 10.3389/fpls.2021.683329 (PMC8293750; doi:10.3389/fpls.2021.683329)
Supplement: Supplementary Figure 1 — The genotype of 132 CSSLs. [file Data_Sheet_1.docx]

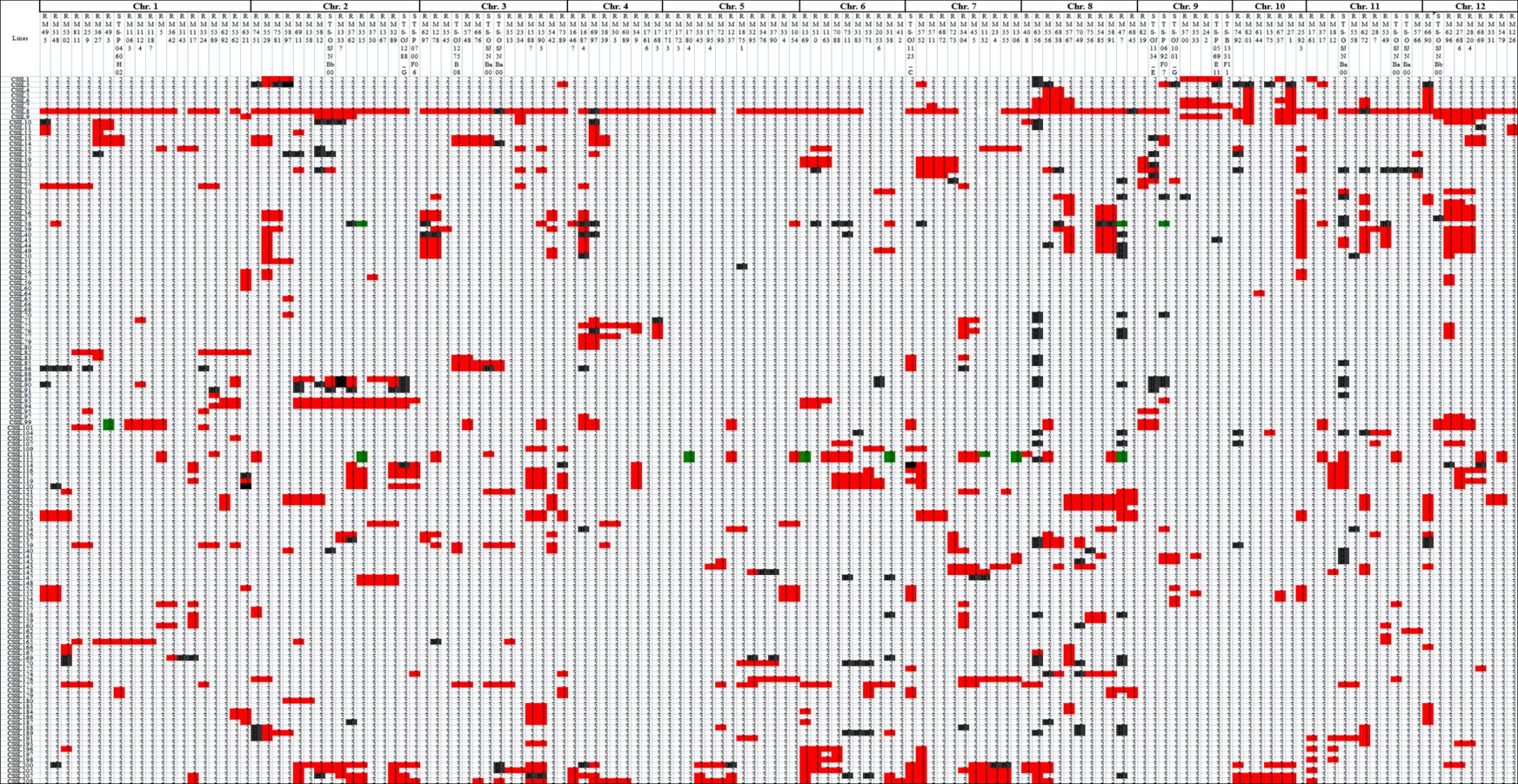


**Figure S1.** The genotype of 132 CSSLs. ‘1’ indicates Koshihikari genotype; ‘2’ represents CH121 genotype; ‘H’ indicates heterozygote; ‘0’ denotes an unknown genotype.

**
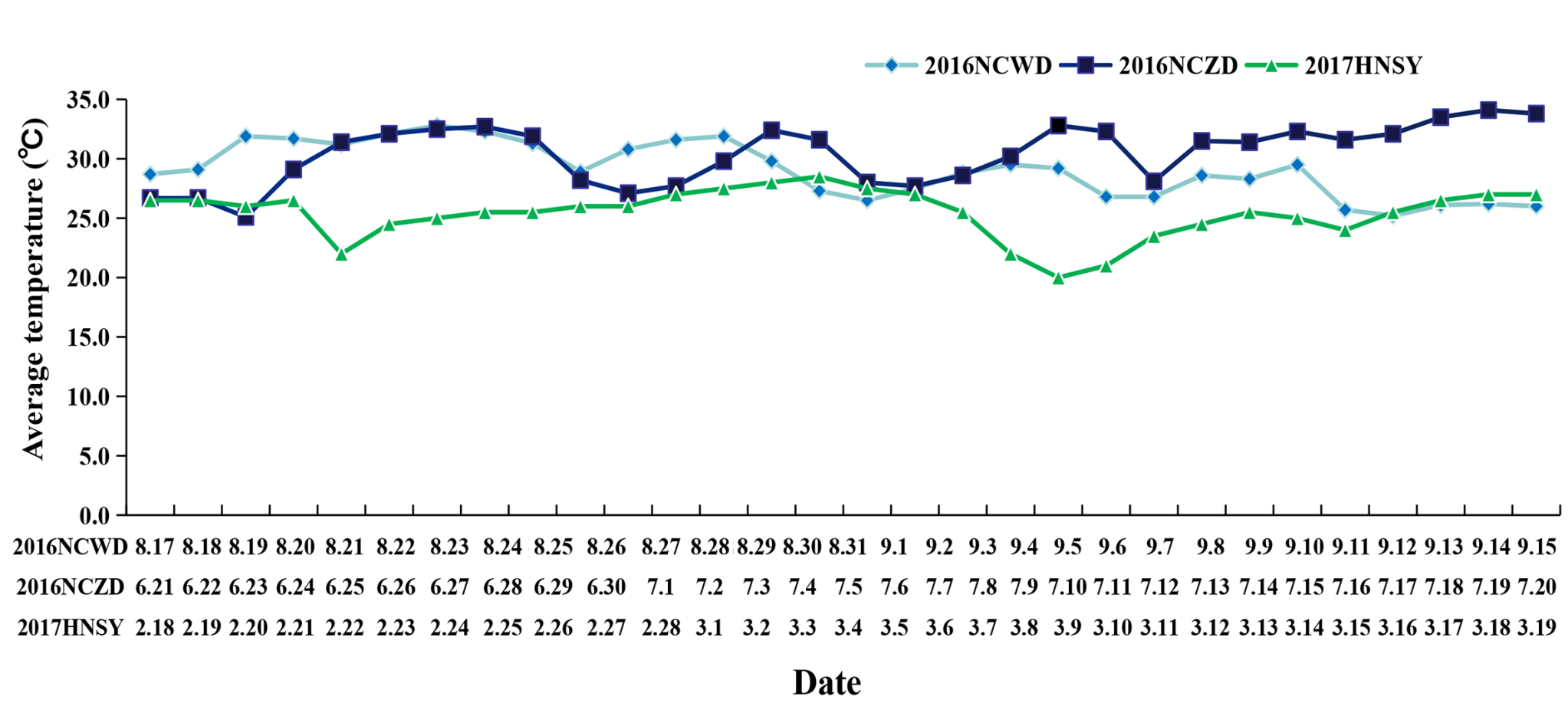
**

**Figure S2.** The daily average temperature of the 30-day period from rice booting to heading stage.

**Table S1** Primers for qRT-PCR in this study

| Primers | Forward(5′-3′) | Reverse(5′-3′) | Type |
| --- | --- | --- | --- |
| *LOC_Os07g41200* | CTCCCCATCTGAACAAGCAG | GAAGTCCAGTGAAGCAGCCT | qRT-PCR |
| *LOC_Os07g41210* | GGCAAGAAGAAAAGGGGAAC | CATTCCCTCCCTTCTGTACG | qRT-PCR |
| *LOC_Os07g41220* | TAGCCGACGTGGATGTTCTG | ATTTTGTGGCGTCGGTTTCG | qRT-PCR |
| *LOC_Os07g41230* | GGACATGATTTTGGCGGTGC | CGCCCTTTGGAGTGTACCAT | qRT-PCR |
| *LOC_Os07g41240* | TCATACAGGAGCACAAGGCG | GCCATGGACGTTTCCTTCTCA | qRT-PCR |
| *LOC_Os07g41250* | GCTGTTTCAGCACCAGTTGATT | TCACAGTCCCCTGCTGAATG | qRT-PCR |
| *LOC_Os07g41260* | TCGAATGGTCCGGGTTTGAG | CATGCAGCAAATACTGGAGCA | qRT-PCR |
| *LOC_Os07g41270* | CCGGTGTTCAAGGAACTCAT | TGCGTGGTAGCTTGGTAGC | qRT-PCR |
| *LOC_Os07g41280* | GATAGCCTGTTTGCTGAATTAC | CTATGGCTCGAAAACTTGACAT | qRT-PCR |

**Table S2** Primers for SSR and InDel markers in this study

| Primers | Forward(5′-3′) | Reverse(5′-3′) | Type |
| --- | --- | --- | --- |
| RM3404 | CTCCTCAGTCCTGAGTCTCCTGTCC | CCCAGAGAGATTACACAGAGCAAGC | SSR |
| RM455 | CCACAAATTAATCCGGATCACACC | AGCATTGTGCAATCACGAGAAGG | SSR |
| RM1132 | TCAAGGTCGACATGTTAGGTATGC | AACCCTATCACCTGAGAAACATCC | SSR |
| RM234 | ACAGTATCCAAGGCCCTGG | CACGTGAGACAAAGACGGAG | SSR |
| RM3555 | TGGAAGTTTCCTGGCGATAG | TGGTTGGACTGAAAAGTCCC | SSR |
| RM1306 | TGCCAATTACCTTCCCGTAC | TGCTCCGTATTGCTGCTATG | SSR |
| P6 | GATAGCCTGTTTGCTGAATTAC | CTATGGCTCGAAAACTTGACAT | InDel |
| P9 | GGCATGATCCATCTACACTTTT | CCTTAAACCCCATGACAAGTAG | InDel |
| P13 | ATGAAACGGTGATGGAGTATGT | AGGAGCCATTTGGAGTTTTACT | InDel |
| P16 | AGAGCGTCTTAGAGACATGCAC | ACTGCTACTTCCACGAAAAAGA | InDel |
| P19 | ACTAACTCCAATCGTCGAAAGA | CTCAATCCTCTTCGCTCTAAAA | InDel |
| P24 | AATCCAACGTTTACAAGGACAG | AGTTGGATGGTTGGATTAGTTG | InDel |
| P25 | GGGGTATTATACGGTGGGTAAT | GAAACGGACTATGCTTTATCGT | InDel |
| P26 | ACGAAGTATTAGCGGAAACAGT | TTTACAAGCGGTTCTCTTATCC | InDel |
| P27 | ACCTTTGCACAAGATCTAAGGA | TTGAAAGTACATCCGTGTGTGT | InDel |

**Table S3** Analysis of variance for aborted spikelets rate of 8 CSSLs in three environments

| SOD | *df* | SS | MS | Variance explained (%) | *F* value |
| --- | --- | --- | --- | --- | --- |
| Line | 7 | 838.39 | 119.77 | 83.63 | 8801.20** |
| Environment | 2 | 57.76 | 28.88 | 5.76 | 2122.08** |
| Line × Environment | 14 | 106.37 | 7.60 | 10.61 | 558.34** |

SOD: Sources of difference. *df*: Degree of freedom. SS: Sum of squares. MS: Mean squares.

‘**’ P < 0.01
